# Intermittent Ventricular Pre‐Excitation: Clinical Features and Electrophysiological Properties

**DOI:** 10.1111/jce.70035

**Published:** 2025-08-07

**Authors:** Antonio Gianluca Robles, Zefferino Palamà, Francesco Santoro, Martin Rauber, Bor Antolič, Domenico Gianfrancesco, Francesco Bartolomucci, Pierluigi Pellegrino, Simona Alfieri, Alessio Borelli, Antonio Scarà, Gabriele De Masi De Luca, Ermenegildo de Ruvo, Leonardo Calò, Matevž Jan, Andrej Pernat, Silvio Romano, Luigi Sciarra

**Affiliations:** ^1^ Cardiology Department L. Bonomo Hospital Andria Italy; ^2^ Department of Clinical Medicine, Public Health, Life and Environmental Science University of L'Aquila L'Aquila Italy; ^3^ Villa Verde C.D.C. Health Centre Taranto Italy; ^4^ Cardiothoracic Department Cardiology Unit, Policlinico Riuniti Foggia Italy; ^5^ Cardiology Department University Medical Centre Ljubljana Ljubljana Slovenia; ^6^ Casa di Cura Di Lorenzo Avezzano Italy; ^7^ Polyclinic Casilino Rome Italy; ^8^ Cardiovascular Surgery Department University Medical Centre Ljubljana Ljubljana Slovenia

**Keywords:** electrophysiological study, intermittent ventricular pre‐excitation, risk stratification, sudden cardiac death, Wolff–Parkinson–White syndrome

## Abstract

**Background:**

Intermittent ventricular pre‐excitation has long been considered a low‐risk marker for sudden death. Accessory pathways (APs) with high‐risk intermittent antegrade conductive properties may exist, but this still represents a gray area in current guidelines. We evaluated differences in risk characteristics between symptomatic and asymptomatic patients with intermittent pre‐excitation (IPX) and those with persistent pre‐excitation (PPX) in a multicenter international registry.

**Methods:**

392 consecutive patients [IPX: 79 (20.15%); PPX: 313 (79.85%)] underwent electrophysiological (EP) study. Data on arrhythmia inducibility (AVRT/AF), AP antegrade conduction properties (ERP/SPERRI), site, and number were collected.

**Results:**

No significant differences were found in demographic characteristics and EP features between PPX and IPX patients, including antegrade conductive properties, prevalence of multiple APs, and AP locations, except for AVRT inducibility which was more frequent in IPX group. Similarly, no differences were detected between symptomatic and asymptomatic IPX patients.

**Conclusions:**

Except for AVRT inducibility, our study shows no significant differences in demographic and other electrophysiological features between PPX and IPX patients. Likewise, no differences in demographic and EP features were detected between symptomatic and asymptomatic IPX patients. Therefore, intermittent pre‐excitation is an unreliable noninvasive marker of arrhythmic risk and it warrants an invasive risk assessment via EP study.

## Graphical abstract

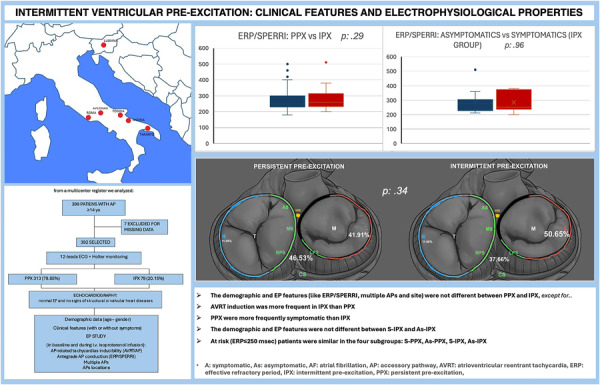

**Abbreviations**
AF: atrial fibrillation
AP: accessory pathway
AS: antero‐septal
AVRT: atrioventricular reentrant tachycardia
CS: coronary sinus
ECG: electrocardiogram
EF: ejection fraction
EP study: electrophysiological study
ERP: effective refractory period
F: female
IPX: intermittent pre‐excitation
LPS: left postero‐septal
M: male
MS: mid‐septal
PPX: persistent pre‐excitation
PS: postero‐septal
RPS: right postero‐septal
SPERRI: shortest pre‐excited RR interval during AF

## 1 Introduction

Symptomatic pre‐excited patients have a clear indication for electrophysiological (EP) study, unlike asymptomatic ones, in whom certain features have historically been used to attempt noninvasive risk stratification [1]. These include AP localization, AP behavior during exercise stress testing (EST), and intermittent loss of ventricular pre‐excitation [2]. The latter, in particular, was long considered a marker of low risk due to its perceived association with poor AP conductive properties [3, 4, 5, 6, 7, 8]. However, some reports have shown that intermittent pre‐excitation can be associated with high antegrade conductive properties and, in some cases, cardiac arrest (CA) [9, 10, 11]. Based on these findings, the latest European guidelines on the management of supraventricular arrhythmias recognize intermittent ventricular pre‐excitation as an imperfect marker of low risk [1], but to date there are scarce studies focused on this topic and no clear indication on how to manage asymptomatic patients with intermittent pre‐excitation.

Catheter ablation of accessory pathways (APs) is recommended in patients with at least one of the following: documented (spontaneous or induced) atrioventricular reentrant tachycardia (AVRT), multiple APs, or rapid antegrade conductive AP properties (defined as ERP or SPERRI ≤ 250 msec at baseline or during intravenous isoproterenol infusion) [1]. The latter is a recognized marker of high risk, necessitating AP ablation due to the potential for CA caused by ventricular fibrillation (VF) triggered by rapidly conducting atrial fibrillation (AF) [1].

While high‐risk markers for catheter ablation are well‐established, reliable noninvasive low‐risk markers, particularly for intermittent pre‐excitation, remain scarce. This study aims to investigate intermittent pre‐excitation, presenting our findings from an international multicenter experience with WPW patients.

## 2 Materials and Methods

### 2.1 Study Population

392 consecutive patients with ventricular pre‐excitation were enrolled in a multicenter registry from August 2005 to April 2024. The enrolling centers were: Policlinico Casilino (Rome, Italy), University of L'Aquila/Casa di Cura Di Lorenzo (Avezzano, Italy), Policlinico Riuniti (Foggia, Italy), Casa di Cura Villa Verde (Taranto, Italy), Ospedale Lorenzo Bonomo (Andria, Italy), and University Medical Center Ljubljana (Slovenia). The study aimed to investigate the clinical and electrophysiological features of ventricular pre‐excitation.

**Figure 1:**
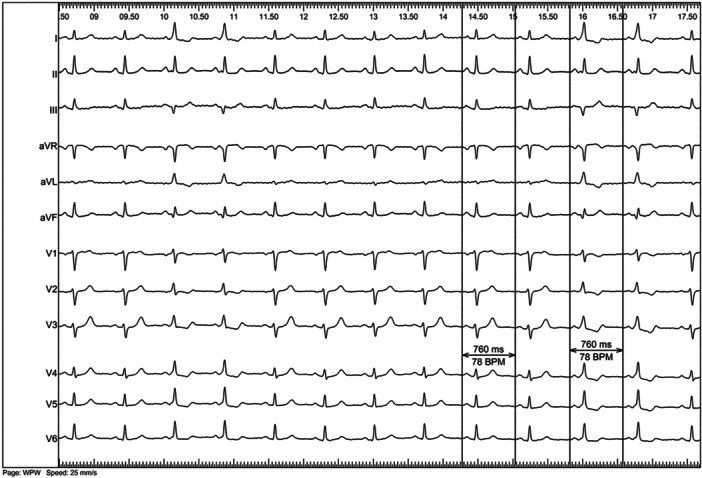
A case of intermittent pre‐excitation in baseline at the beginning of an EP study. All the QRS complexes are narrow, but the 3°, 4° and the 11°, 12°, are wide and show an initial δ wave. δ wave is positive in V1, negative in D3‐aVF, and positive in D1‐aVL thus suggesting a left‐sided postero‐septal AP (confirmed by 3D‐mapping). The majority of beats abruptly show normal PQ, no δ wave and narrow QRS with normal morphology (physiological septal activation testified by the small q wave in V5–V6). This is a teaching case of intermittent pre‐excitation because of the abrupt disappearance of δ wave is not related to heart rate variation (the heart rate is fixed at 78 bpm as confirmed by mesaurement of P‐P cycle lenght) or isoproterenol infusion.

Patients enrolled were both symptomatic and asymptomatic. Symptoms were defined as palpitations reported during the outpatient visit, regardless of AVRT/AF recording before EP study. Based on the preprocedural evaluation represented by 12‐lead ECG and/or Holter monitoring, patients were divided into two groups: those with intermittent pre‐excitation (IPX) and those with persistent pre‐excitation (PPX). IPX was defined as the sudden loss of delta wave at resting 12‐lead ECG and/or at Holter monitoring, not related to heart rate modifications or ongoing treatment with antiarrhythmic drugs (AADs). Figure 1 shows an example of IPX.

**Figure 2:**
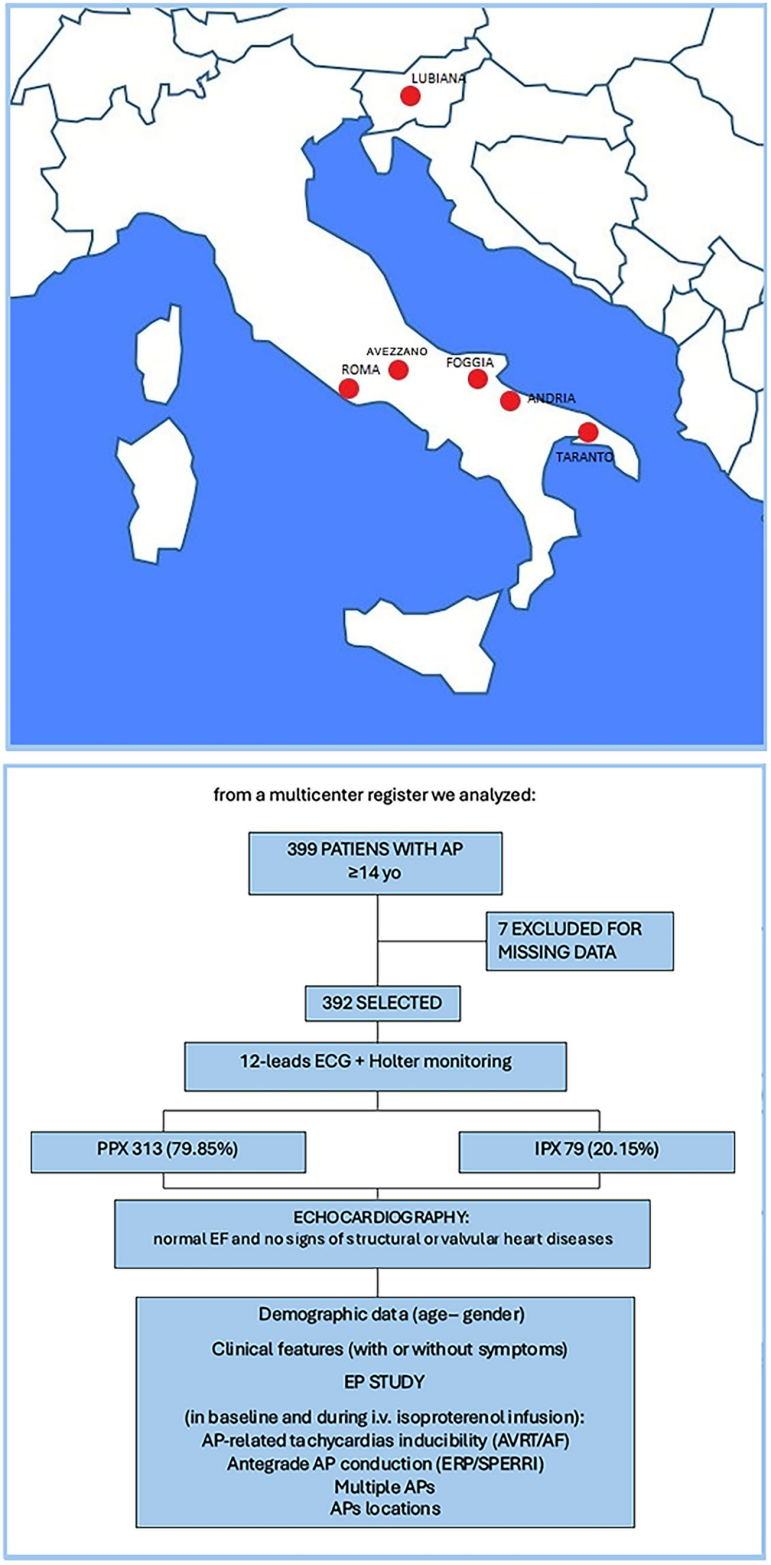
Study design synopsis.

In total, 313 patients had PPX (79.85%) and 79 had IPX (20.15%). Each patient underwent transthoracic cardiac ultrasound evaluation, and all had normal ejection fraction (EF) and no signs of structural or valvular heart diseases. All patients provided written informed consent before undergoing EP study and for the use of their data for scientific purposes. The study was carried out under the Declaration of Helsinki. Figure 2 summarizes the study design.

### 2.2 EP Study

Each electrophysiology (EP) lab had its own digital recording system, and the settings depended on the center and operators' preferences. No EP study was conducted under general anesthesia or deep sedation. A detailed description of center‐specific EP lab settings, materials used (including the use of three‐dimensional mapping systems), and energy sources (radiofrequency or cryo‐energy) is beyond the scope of this paper.

The EP study workflow included ventricular programmed stimulation, atrial programmed stimulation, atrial incremental pacing and atrial bursts. All these maneuvers were also performed during intravenous infusion of isoproterenol (0.1 μg/kg bolus over 1 min, followed by a 0.01 − 0.02 μg/kg/min infusion) if AVRT/AF were not inducible under basal conditions. If pre‐excited AF was induced, SPERRI was measured in baseline and after i.v. isoproterenol administration. If arrhythmias were not inducible, then APs' ERP was measured in baseline and after i.v. isoptoterenol infusion. To facilitate comparative analysis, we reported the shortest coupling interval representing the maximal antegrade conductive property over the AP for each patient. Specifically, this was represented by the only shortest ERP or SPERRI, at baseline if already ≤ 250 msec (at risk) or alternatively under intravenous isoproterenol infusion if > 250 msec (not at risk) at baseline [1].

During EP study and mapping, AP locations were identified and annotated, with attention to multiple APs. Multiple accessory pathways were defined as the presence of two or more pathways separated by at least 1–3 cm [12]. Patients with multiple APs were excluded from inter‐ and intra‐group analyses to avoid bias during the comparison of AP site distribution.

**Figure 3:**
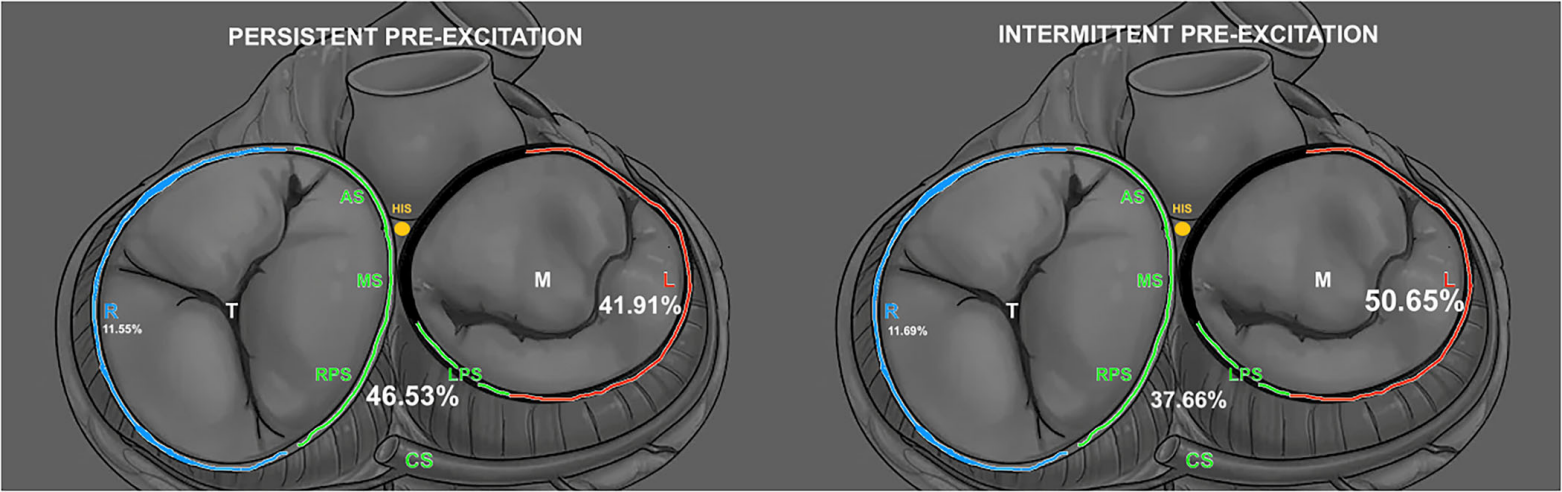
APs site distribution. R, right‐sided; L, left‐sided; AS, antero‐septal; MS, Mid‐septal; RPS, right postero‐septal; LPS, Left postero‐septal; CS, coronary sinus; T, tricuspid valve; Mitral valve. Notably, septal AP include AS, MS, RPS, proximal CS and LPS.

AP locations were classified as follows: right free‐wall (right‐sided), left free‐wall (left‐sided), and septal [including antero‐septal, mid‐septal, right‐, left‐postero‐septal, and proximal coronary sinus (CS)] (Figure 3). Their anatomical differentiation has been previously described [13].

If AVRT and/or rapid‐conducting pre‐excited AF were non‐inducible, AP ablation was eventually performed in cases of multiple APs or if the ERP was ≤ 250 msec [1].

### 2.3 Statistical Analysis

Continuous variables were expressed as the mean ± standard deviation (SD) value. The differences in the continuous variables between groups were compared by *t*‐test per data with normal distribution. Categorical variables were expressed as numbers and percentages and their differences were investigated with a Chi‐square test (with Yates correction when appropriate) or Fisher's exact test. *p* < 0.05 was considered statistically significant.

## 3 Results

Table 1 and Table 2 represent results in detail.

**Table 1:** Demographic, clinical and electrophysiological features: comparison between the two groups (IPX vs. PPX). M, male. *p* significative at < 0.05.

	PPX 313 (79.85%)	IPX 79 (20.15%)	*p* value
Age	37.08 + / − 16.27	38.15 + / − 16.30	0.59
Gender	M 203 (64.85%)	M 44 (55.70%)	0.13
ERP/SPERRI	268.00 + / − 58.39 msec	282.08 + / − 58.89 msec	0.29
◄ ≤ 250 msec	63 (20.13%)	11 (13.92%)	0.27
◄ M ≤ 250 msec	47 (74.60%)	5 (45.45%)	0.43
Symptomatic	304 (97.12%)	70 (88.60%)	< 0.001
Inducibility:			
Any of AVRT and/or AF	134 (42.81%)	52 (65.82%)	< 0.001
AVRT only	96 (30.67%)	37 (46.83%)	< 0.01
AF only	21 (6.71%)	10 (12.66%)	0.08
AVRT + AF	17 (5.43%)	5 (6.33%)	0.78
AP site:			0.34
▪ Left	127 (41.91%)	39 (50.65%)	0.17
▪ Right	35 (11.55%)	9 (11.69%)	0.97
▪ Septal	141 (46.53%)	29 (37.66%)	0.16
Multiple APs	10 (3.19%)	2 (2.53%)	1

**Table 2:** Demographic and electrophysiological features: comparison between symptomatics and asymptomatics in the IPX cohort. M, male. *p* significative at < 0.05.

	Symptomatic 70 (88.61%)	asymptomatic 9 (11.39%)	*p* value
Age	37.69 + / − 16.33	41.78 + / − 16.02	0.42
Gender	M 41 (58.54%)	M 3 (33.33%)	0.17
ERP/SPERRI	281.67 + / − 58.67 msec	283.33 + / − 60.22 msec	0.96
Inducibility:			
Any of AVRT and/or AF	47 (67.14)	5 (55.55)	0.48
AVRT only	38 (54.28%)	4 (44.44%)	0.73
AF only	9 (12.86%)	1 (11.11%)	1
AVRT + AF	5 (7.14%)	0 (0%)	1
AP site:			0.18
▪ Left	32 (45.71%)	7 (77.78%)	0.14
▪ Right	8 (11.43%)	1 (11.11%)	0.59
▪ Septal	28 (40%)	1 (11.11%)	0.18
Multiple APs	2 (2.86%)	0 (0%)	1

### 3.1 Study Population

In the sample of 392 WPW patients, 313 (79.85%) had PPX and 79 (20.15%) had IPX. There were no differences between PPX and IPX in terms of age at the time of electrophysiological (EP) study (37.08 ± 16.27 vs. 38.15 ± 16.30 years, respectively; *p* = 0.59) and gender (male: 203 [64.85%] vs. 44 [55.70%], respectively; *p* = 0.13). Interestingly, the manifestation of symptoms was found to be statistically different between the two groups, revealing that PPX patients were more often symptomatic (304 [97.12%] vs. 70 [88.60%], respectively; *p* < 0.001).

### 3.2 EP Study Findings

**Figure 4:**
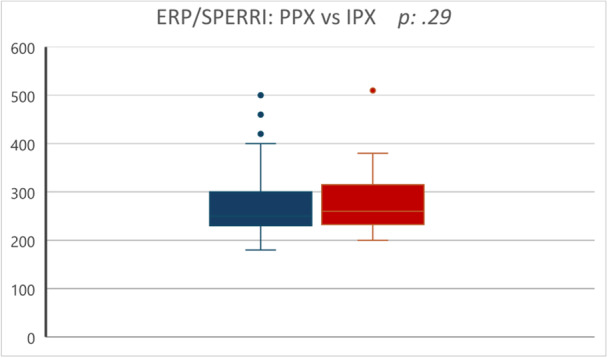
Box plot: intergroup comparison (PPX vs. IPX) of AP antegrade conductive properties (shortest ERP/SPERRI).

There were no statistically significant differences in APs' antegrade conductive properties between PPX and IPX (shortest ERP/SPERRI: 268 ± 58.39 msec vs. 282.08 ± 58.89 msec, respectively; *p* = 0.29) (Figure 4). Conversely, the global inducibility of arrhythmias related to AP (AVRT/AF) showed statistically significant differences (PPX: 134 [42.81%] vs. IPX: 52 [65.82%]; *p* < 0.001), similarly AVRT was slightly more frequently inducible in IPX (PPX: 96 [30.67%] vs. IPX: 37 [46.83%]; *p* < 0.01).

Multiple accessory pathways were found in 10 patients in the PPX group (3.19%) and in 2 patients in the IPX group (2.53%), both of whom were females (100%), with no statistically significant differences (*p* = 1). There were 303 patients in the PPX group with a single AP, distributed as follows: 35 right‐sided, 141 septal, and 127 left‐sided. In the IPX group, there were 77 patients with a single AP, distributed as follows: 9 right‐sided, 29 septal, and 39 left‐sided. The AP site distribution in both groups did not show statistically significant differences (*p* = 0.34) (Figure 3).

### 3.3 Clinical and Electrophysiological Features of Intermittent WPW Patients

In the IPX group, there were 70 (88.61%) symptomatic patients and 9 (11.39%) asymptomatic patients, with no differences in terms of age (37.69 ± 16.33 vs. 41.78 ± 16.02 years; *p* = 0.42) and gender (male: 41 [58.54%] vs. 3 [33.33%]; *p* = 0.17). There were also no statistically significant differences in electrophysiological features between symptomatic and asymptomatic patients in the IPX group.

**Figure 5:**
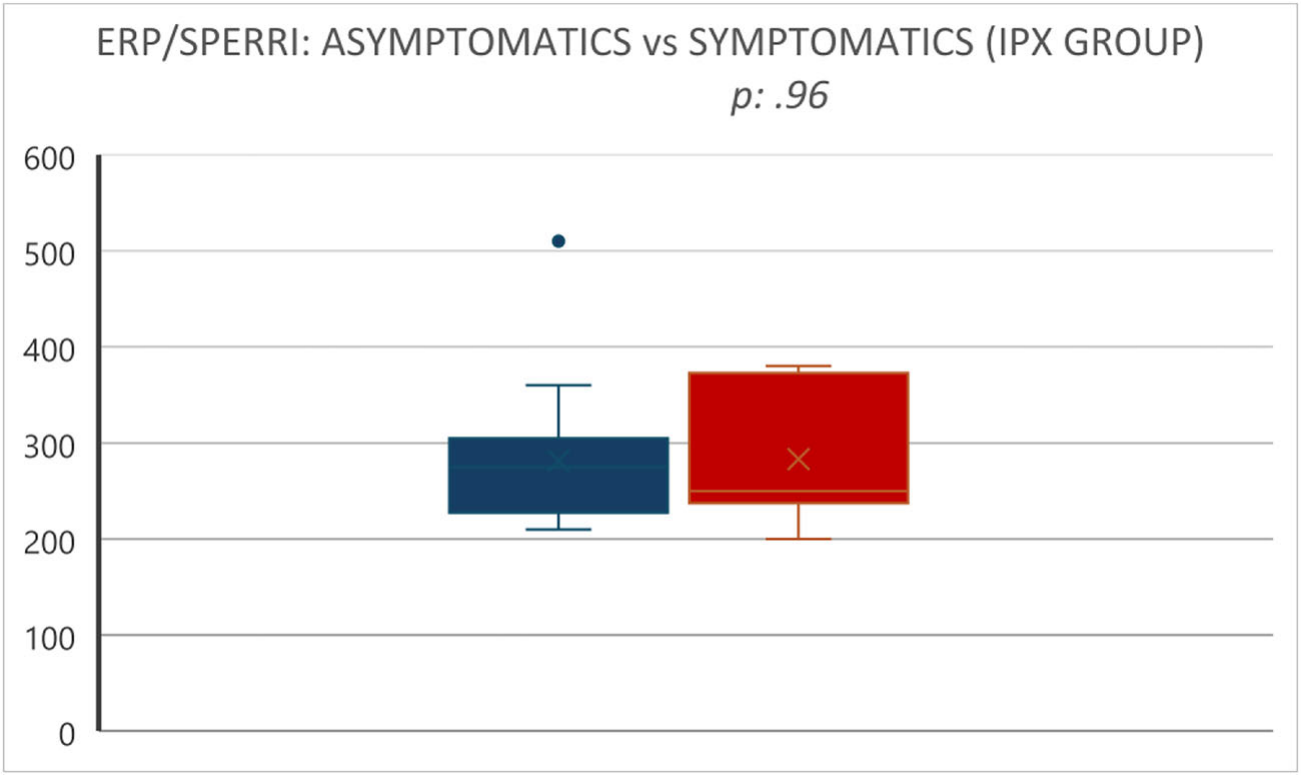
Box plot: intragroup comparison (symptomatic IPX vs. asymptomatic IPX) of AP antegrade conductive properties (shortest ERP/SPERRI).

In particular, the shortest ERP/SPERRI was 281.67 ± 58.67 msec versus 283.33 ± 60.22 msec, respectively (*p* = 0.96) (Figure 5). AVRT/AF inducibility was observed in 47 (67.14%) symptomatic patients versus 5 (55.55%) asymptomatic patients (*p* = 0.48).

There were 68 patients in the symptomatic IPX group with a single AP, distributed as follows: 8 right‐sided, 28 septal, and 32 left‐sided. In the asymptomatic IPX group, all 9 patients had a single AP, distributed as follows: 1 right‐sided, 1 septal, and 7 left‐sided. The AP site distribution in both subgroups did not show statistically significant differences (*p* = 0.18). Two patients with multiple APs were found in the symptomatic IPX group, while none were found in the asymptomatic IPX group (*p* = 1).

### 3.4 ERP/SPERRI Intergroup Comparison

Finally, the analysis of APs' antegrade conductive properties deserves specific mention. In particular, when divided into shortest ERP/SPERRI ≤ 250 msec (at risk) vs. shortest ERP/SPERRI > 250 msec (not at risk), the four subgroups (symptomatic persistent, asymptomatic persistent, symptomatic intermittent, and asymptomatic intermittent) did not show significant differences (*p* = 0.67) (Table 3).

**Table 3:** Intergroup comparison of antegrade conductive properties. When splitted for ERP/SPERRI ≤ 250 msec (at risk) and ERP/SPERRI > 250 mses (non at risk) the four groups appeared to be homogenous.

	S‐PPX	As‐PPX	S‐IPX	As‐IPX	
ERP/SPERRI ≤ 250 msec	59 (50%)	4 (50%)	7 (38.89%)	4 (66.67%)	p. 0.67
ERP/SPERRI > 250 msec	59 (50%)	4 (50%)	11 (61.11%)	2 (33.33%)	

Abbreviations: As, asymptomatic; ERP, effective refractory period; IPX, intermittent pre‐excitation; PPX, persistent pre‐excitation; S, symptomatic; SPERRI, shorter pre‐excited RR interval during AF.

## 4 Discussion

### 4.1 Main Findings

We present one of the largest multicenter European registries of patients with ventricular pre‐excitation. Key findings are: (1) no differences in terms of demographic features and electrophysiologic properties between patients with persistent and intermittent ventricular pre‐excitation, except for AVRT inducibility at AP study, (2) demographic and electrophysiologic characteristics were not different between symptomatic and asymptomatic intermittent ones, (3) in particular, the four subgroups of symptomatic persistent, asymptomatic persistent, symptomatic intermittent, and asymptomatic intermittent appeared similar for the risk established according to ERP/SPERRI cut‐off 250 msec.

Our findings are consistent with the previous study of our group, which demonstrated no statistically significant differences between demographic and electrophysiologic features between PPX and IPX from a sample of symptomatic adults ( ≥ 18 years old) [14]. In the present study, the increased likelihood of symptoms in PPX patients may be related to the longer duration of pre‐excitation, thus PPX patients have a greater temporal activation burden of the accessory pathway than patients with IPX, and therefore the onset of an arrhythmia that causes palpitations is statistically more likely over time. But this is in contrast with the greater inducibility of AVRT we found in intermittent ones. This can be explained by invoking the retrograde conduction properties of an intermittent pathway that most often fails to conduct an atrial extra beat anterogradely but remains viable in the opposite direction and may facilitate by this mechanism the initiation of an orthodromic AVRT.

Indeed, even if a detailed description and analysis of retrogade APs' properties is beyond the aims of this study, looking at our data of intermittent sample, we saw that patients more prone to AVRT inducibility had rouglhy higher antegrade ERP. Probably, an alternative explation of the greater prevalence of symptoms in PPX than IPX may be related to patients' perception: being aware of costant pre‐excitation may allert them and consequently alter their perception. This underlines how it could be imperfect classifing a patient as “symptomatic” basing only on palpitations reporting and not on arrhythmia recordings. We should also bare in mind that asymptomatic pre‐excited patients may develop symptoms over time, even if having symptoms after 40 years old is unlikely [15, 16].

Results of demographic and electrophysiologic evaluation are clear: the two populations appear homogeneous, and the demographic characteristics of age and gender did not show statistically significant differences between the two groups. Likewise, it was for the antegrade conduction properties (shortest ERP/SPERRI), AVRT/AF inducibility, as well as the number of APs and their location. Even if in absolute terms APs responsible for IPX were found to be more left‐sided (50%; vs. 41% in the PPX group) rather than septal (37%; vs. 46% in the PPX group), this difference was not statistically significant. Probably, the left atrial location of an AP is not enough to explain its intermittent manifestation, particularly by invoking the theory that greater anatomical distance from the sinus node favors conduction along the AV node‐Hisian pathway and consequently a less marked pre‐excitation [14].

In the intermittent group only, there were nonsignificant differences in demographic and electrophysiological features. These results are in line with the recently published study by Yammine et al. [17]. They did not find differences between clinical and electrophysiological features between symptomatic and asymptomatic children with intermittent pre‐excitation, except for AVRT inducibility (resulted greater in the symptomatic) and ERP under stress condition (resulted greater in the asymptomatic) [17]. The latter may strenghten the concept that asimptomaticity alone does not exclude high risk APs in young people and may contrast the previous idea by Pappone et al. supporting inducibility—which is more relatable to symptoms—as a better predictor of future events than ERP alone [18, 19]. In our study, instead, the absence of differences may be due to the low number of intermittent asymptomatic and the wider age spectrum considered, including mostly adults.

### 4.2 Noninvasive Risk Stratification: Is It Enough?

Based on these findings, we conclude that the presence alone of an IPX does not exclude the possibility of high‐risk features at electrophysiological (EP) studies, such as inducibility, rapid anterograde conductive properties, and multiple accessory pathways. These findings are consistent with previously published papers on pediatric or young populations ( < 21 years old) [20, 21, 22].

In the past, abrupt loss of pre‐excitation at EST was considered a reliable marker of low risk, as suggested by Levy et al. [15], but a recent prospective study by Jemtrén et al. demonstrated a low negative predictive value of exercise stress testing in excluding high‐risk APs in both symptomatic and asymptomatic patients [23]. Moreover, a recent study by Yammine et al. suggests that noninvasive risk stratification can fail to identify children with IPX at risk of life‐threatening events, concluding that all children with ventricular pre‐excitation should undergo EP study regardless of symptoms [17].

According to the ESC 2019 guidelines [1], an ERP ≤ 250 msec is considered indicative for a high‐risk AP, which ‐ from a physiological perspective ‐ would require a sinus rate > 240 bpm and it is unlikely to be achieved during exercise stress testing (EST), particularly in adults. However, this presents a paradox, as the maximum achievable sinus rate can be estimated using the well‐known formula 220 ‐ age. Furthermore, to reliably identify a low‐risk AP, at EST should disappear pre‐excitation and at the same time appear such a grade of atrio‐ventricular block. In contrast, the high heart rates required to assess AV conduction can be reached during spontaneous or evoked AVRT/AF or incremental atrial pacing, especially during IV isoproterenol administration. This highlights the higher accuracy and negative predictive value of EP study than EST [15, 17, 23].

In summary, there are limitations to noninvasive risk stratification of ventricular pre‐excitation in asymptomatic patients, especially in young individuals. Natural history studies on WPW have shown that cardiac arrest is more frequent in young ( < 40 years old) and symptomatic patients, but it also depends on electrophysiological properties of the APs, such as inducibility (especially orthodromic AVRT) and low antegrade conductive properties (ERP ≤ 250 msec) [6, 15, 16, 24, 25, 26]. Our findings strongly support the use of invasive risk assessment in asymptomatic and/or intermittent pre‐excited patients, who were previously considered a low‐risk population and not worthy of EP study.

### 4.3 Limitations

The main limitation of this study is the low number of asymptomatic patients, particularly in the intermittent group, whose characteristics are crucial for infer on arrhythmic risk stratification. Another potential limitation is the imperfect classification of “symptomatic” patients, defined as those experiencing palpitations with or without documented arrhythmias. Additionally, combining ERP and SPERRI into a single category for anterograde conductive properties may be limiting, as these parameters are not equivalent. Moreover, the nonavailability of APs' ERP for each patient represents a limit, but this depends on the already described workflow and it reflects heterogeneity over time between operators and centers.

However, this study aimed to define clinical and electrophysiological features of patients with intermittent ventricular pre‐excitation compared to those with persistent pre‐excitation, using an international multicenter registry mostly made up by retrospective data. We also performed an intragroup analysis comparing symptomatic and asymptomatic intermittent patients. In these cohort we focused in particular on arrhythmia inducibility, *at risk* ERP/SPERRI and prevalence of multiple APs (guidelines criteria for mandatory AP ablation) to rebut the idea that intermittent pre‐excitation is a low arrhythmic risk marker.

Estimating arrhythmic risk and understanding the natural history of intermittent pre‐excitation were beyond the scope of this study, which instead provides a foundation for future larger and prospective studies powered ad hoc to investigate this underexplored topic. As previously noted, such studies would be ideal but are challenging to conduct.

## 5 Conclusions

In this multicenter cohort of patients with ventricular pre‐excitation, we found no significant differences in demographic and electrophysiological features between those with IPX and those with PPX, except for AVRT inducibility which was more frequent in IPX group. Similarly, no differences were observed between symptomatic and asymptomatic IPX patients. Given that IPX is an unreliable marker of arrhythmic risk, invasive EP study should be considered in IPX patients, regardless of symptoms, for a more accurate risk evaluation.

## Conflicts of Interest

The authors declare no conflicts of interest.

## Data Availability

Data are available upon request to the corresponding author.
